# The Effect of Red and Blue on Gross and Fine Motor Tasks: Confirming the Inverted-U Hypothesis

**DOI:** 10.3389/fpsyg.2021.744913

**Published:** 2022-01-06

**Authors:** Xiaobin Hong, Aiai Xu, Yan Shi, Lu Geng, Rong Zou, Yuanbing Guo

**Affiliations:** ^1^Hubei Key Laboratory of Exercise Training and Monitoring, College of Health Science, Wuhan Sports University, Wuhan, China; ^2^Department of Psychology, Wuhan Sports University, Wuhan, China

**Keywords:** color effect, red, blue, arousal, dart-throwing, grip strength

## Abstract

Previous studies have shown that the color red can affect basic motor functioning. However, these studies utilized simple gross motor tasks rather than those assessing complex fine motor skills. Moreover, these empirical studies were theoretically based on the threat–behavior link in human and non-human animals, and neglected the relationship between arousal and motor performance. According to the Yerkes–Dodson law and the inverted-U hypothesis in sport psychology, for simple motor tasks, high arousal (associated with the color red) is more advantageous than low arousal (associated with the color blue); for complex motor tasks, low arousal (blue color) is more advantageous than high arousal (red color). The current research examined the effect of color on different kinds of motor skills (fine motor and gross motor) based on the inverted U-hypothesis. In Experiment 1, we examined the effect of red and blue on dart-throwing performance, whereas in Experiment 2, we examined the effect of red and blue on grip strength performance. The results showed that performance of fine motor skill (dart-throwing) in the blue condition was better than in the red condition, and performance of gross motor skill (handgrip) in the red context was better than in the blue context. These results indicate that the type of motor skill assessed moderates the influence of red and blue on motor performance.

## Introduction

Color is perceived visually and has been found to affect psychological function. Over the past decades, a variety of studies have examined the psychological effects of color, and most have focused on the color red, which has been explored in widely different domains. For example, some studies have found that red undermined performance on tests assessing intelligence as compared with blue in academic contexts (Mehta and Zhu, 2009). Other studies have found that red increases individual attraction in relational contexts (Elliot and Niesta, 2008). Further domains in which color has been studied include driving, dieting, financial prediction, and competitive sports (Hill and Barton, 2005; Guéguen et al., 2012; Bruno et al., 2013; Jiang et al., 2014).

In competitive sports, Hill and Barton (2005) reported that athletes wearing red uniforms had a higher ratio of wins as compared with athletes wearing blue uniforms. This work provoked further interest in testing the advantages of wearing red uniforms although subsequent findings have been inconsistent (Attrill et al., 2008; Sorokowski and Szmajke, 2011; Piatti et al., 2012; Curby, 2016). One explanation for these inconsistent findings might be that many factors may affect the performance of athletes in competition, and the impact of color in this context might not be strong enough. To explore the psychological effect of color in competitive sports more thoroughly, it is necessary to investigate the effect of red on motor behavior. While there are many studies on the relationship between red and cognitive performance in academic contexts, the relationship between red and motor behavior remains poorly studied.

In terms of the relationship between color and motor behavior, older studies have examined the effect of color on muscular strength (Green et al., 1982; Ingram and Lieberman, 1985; Profusek and Rainey, 1987; Crane et al., 2008). However, due to the lack of an explicit theoretical basis and methodological problems, these results were also inconsistent. Taking into account the threat–behavior link, Elliot and Aarts (2011) examined the effect of the color red on basic motor functioning and showed that red improves strength output and velocity of force. Payen et al. (2011) designed an experiment in which participants had to perform a maximal muscle contraction while viewing either color red, a chromatic control color, or an achromatic control color. Results showed increased strength while viewing red but no significant difference in the peak amplitude of power. Dreiskaemper et al. (2013) created an artificial fighting situation to examine the influence of red jerseys on physical parameters in combat sports. Results showed that participants in red jerseys had significantly higher heart rates and significantly higher pre-contest values on the strength test. However, these studies only examined the effect of red on gross motor skill performance (e.g., a strength task). What effect does the color red have on fine motor skill performance? Elliot and Aarts (2011) argued that future work should focus on whether red facilitates certain types of overt actions more than others. In addition, Elliot and Aarts (2011) only used the red-threat link to explain why red improved basic motor performance, and did not consider the relationship between emotional arousal and performance.

Although the relationship between color and emotional arousal is still debated, it is generally believed that long-wave colors (e.g., red) can cause higher arousal than short-wave colors (e.g., blue) (Wexner, 1954; Wright and Rainwater, 1962; Jacobs and Suess, 1975; Walters et al., 1982; Stone, 2003). Red is commonly associated with danger and aggression, and blue is commonly associated with tranquility, meaning that red may cause higher emotional arousal than blue. According to the Yerkes–Dodson law, for simple tasks, high arousal is preferable to low arousal; for difficult tasks, low arousal is preferable to high arousal due to the effects on performance (Yerkes and Dodson, 1908). Based on an analysis of previous studies, Xia et al. (2016) argued that the influence of red and blue on cognitive tasks may be modulated by the difficulty of the tasks due to the relationship between color and emotional arousal. They examined the effect of color on cognitive performance while manipulating the difficulty and type of task. The results showed that the difficulty of the task moderated the relationship between red and performance on a detail-oriented task, while blue enhanced performance on both a simple and difficult creative task. We postulate that creative tasks are difficult tasks (as compared with detail-oriented tasks), and as such, high arousal would not help performance.

The results of Xia et al. (2016) supported the assertion that the relationship between color and cognitive performance is influenced by emotional arousal. For motor tasks, is the effect of color on motor performance related to emotional arousal? Compared with gross motor skills, fine motor skills involve very precise movements which are accomplished using smaller muscles. On the difficulty–simplicity dimension, fine motor skills might be required for difficult motor tasks, while gross motor skills might be required for simple motor tasks. Therefore, the relationship between color and motor performance is consistent with the Yerkes–Dodson law. Moreover, the inverted-U hypothesis in sport psychology also indicates that optimal arousal depends on the type of motor task (Williams, 1994). Specifically, the optimal arousal for gross motor skills is higher than for fine motor skills (Oxendine, 1970).

On this basis, we conducted two experiments to further explore the effect of color (red versus blue) on different types of motor performance. Experiment 1 examined the effect of red/blue on fine motor skill (dart-throwing) performance and Experiment 2 examined the effect of red/blue on gross motor skill (handgrip) performance. Given the documented relationship between color and arousal, as well as arousal and motor performance, we predicted that blue would enhance fine motor skill (dart-throwing) performance, and red would improve gross motor skill (handgrip) performance. In addition, we also assessed anxiety and self-efficacy as process variables to explore these as possible mediators of the relationship between color and motor performance.

## Experiment 1

The aim of Experiment 1 was to examine the influence of red versus blue on dart-throwing performance, anxiety, and self-efficacy.

### Materials and Methods

#### Participants

Participants were 34 male college students and graduate students (age range = 19–25 years) who were right-handed. The study protocol was approved by the Wuhan Sports University Medical Ethics Committee. Written informed consent was obtained from each participant, and participants could freely withdraw at any time. All participants were healthy and none was color blind.

#### Materials and Procedure

Participants came into the research laboratory individually and the researcher explained the dart-throwing task and scoring method. Next, participants were directed to stand behind a throwing line that was positioned 237 cm from the wall in front of a standard dartboard that was mounted at a height of 173 cm. Participants were free to practice for 5 min (about 20 warm-up darts). After the warm-up, participants were instructed to perform 20 dart-throws in the control condition (white wall) as the baseline measure. Then, all participants completed 20 dart-throws in red (laboratory A) and blue (laboratory B) conditions separately. Participants took about 5 min to complete the 20 trials in each condition. During this period, participants adjusted each throwing movement and completed all the throwing process. To avoid sequence effects, half the participants completed 20 dart-throws in the red condition and then completed 20 dart-throws in the blue condition. The remaining half completed the conditions in reverse order. In laboratory A, the dartboard was hung on a red wall which was 2 m long and 1.5 m wide. The dimensions of laboratory B were identical but the dartboard was hung on a blue wall. The temperature of each laboratory was 20°C, and each was moderately bright and soundproof. The RGB criteria of red (255, 0, and 0) and blue (0, 0, and 255) were applied to the background wall. The HSL (hue, saturation, and lightness) criteria were as follows: red (0, 240, and 120) and blue (160, 240, and 120). When participants completed 20 dart-throws in the red or blue condition, they were asked to close their eyes for 3 min to eliminate visual after-sensation from the color.

The dartboard was an 18-inch international standard board with black and white on the reverse side. To avoid the influence of the red bull’s-eye, it was changed to black. The darts were made of pure copper and weighed 25 grams. They were a metallic color. Darts that hit the bull’s-eye were awarded 10 points, darts that hit the circle adjacent to the bull’s-eye were awarded 9 points, and darts that hit the next adjacent circle were awarded 8 points, and so on. Darts that missed the dartboard completely were awarded 0 points.

After completing the first 10 darts, two assessments were administered sequentially. Participants were asked to complete measures of anxiety and self-efficacy. A single item was used to assess anxiety (“I am anxious about the task”) and another to assess self-efficacy (“I will perform my best in the next task”) on a 9-point scale, from 1 (“not at all true of me”) to 9 (“very true of me”).

#### Statistical Analysis

Firstly, all data were tested for normal distribution to examine whether they were conformed to normal distribution. Secondly, to test the influence of red versus blue on dart-throwing performance, we performed analysis of covariance (ANCOVA) to control for baseline performance. The dependent measure was the total score across the 20 trials. Thirdly, paired-samples *t*-tests were used to compare anxiety and self-efficacy in the red and blue background conditions.

### Results and Discussion

A sample K-S analysis proved that the data was a normal distribution (*Z* = 0.067, *P* = 0.200). A repeated measures ANCOVA revealed a significant effect of color background condition, *F*(1, 67) = 8.61, *p* = 0.005 (*p* < 0.01), η^2^ = 0.12. Dart-throwing performance was higher in the blue background condition (*M* = 133.29, *SD* = 15.87) than the red (*M* = 124.53, *SD* = 10.84), consistent with the hypothesis that blue would enhance the performance of fine motor skill (i.e., dart-throwing). Thus, it appears that background color had a differential effect on motor performance as the high arousal induced by red had a minimal positive influence on fine motor skill whereas the low arousal induced by blue was beneficial.

Paired-samples *t*-tests showed significant differences for anxiety in the different color conditions, *t* = 3.65, *p* = 0.001, Cohen’s *d* = 0.3929(a small to medium effect size for anxiety; Cohen, 1988). Participant anxiety was higher in the red condition (*M* = 3.59, *SD* = 2.35) than the blue (*M* = 2.71, *SD* = 2.07). There was also a significant difference for self-efficacy, *t* = –2.72, *p* = 0.01, Cohen’s *d* = 0.427(a nearly medium effect size for self-efficacy; Cohen, 1988). Participant self-efficacy was higher in the blue background condition (*M* = 7.24, *SD* = 1.52) than the red (*M* = 6.56, *SD* = 1.66).

In order to further explore whether anxiety and self-efficacy influenced performance independent of color condition, we conducted a mediational analysis on the results of Experiment 1. An indirect effect of color on motor performance via anxiety was not significant (*B* = −0.29, *p* > 0.05), and the bootstrapping revealed the null mediation effects (95% CI: [−0.12, 0.07]). The above interval contained 0, which indicated that the mediating effects of anxiety were not significant. An indirect effect of color on motor performance via self-efficacy was also not significant (*B* = −0.41, *p*> 0.05), and the bootstrapping revealed the null mediation effects (95%CI: [−0.16, 0.08]). The above interval contained 0, which indicated that the mediating effects of self-efficacy were also not significant. Although the results of dart-throwing performance confirmed the inverted-U hypothesis, null mediation of anxiety and self-efficacy indicated that anxiety and self-efficacy may be not strong contributors to dart-throwing performance.

## Experiment 2

The aim of Experiment 2 was to examine the influence of red versus blue on handgrip performance, anxiety, and self-efficacy. Experiment 2 used a between-subject color manipulation and different procedure than Experiment 1 based on a study by Elliot and Aarts (2011).

### Materials and Methods

#### Participants

A total of 64 male college students and graduate students were recruited from Wuhan, China (age range 18–25 years). All participants were male, right-handed, and with no experience in combat sports or weight lifting. They were instructed to refrain from participating in any rigorous physical activity or consuming caffeine prior to the experimental session. The study protocol was approved by the Wuhan Sports University Medical Ethics Committee. Written informed consent was obtained from each participant, and participants could freely withdraw at any time. All participants were healthy and none was color blind.

#### Materials and Procedure

A total of 64 participants were randomly assigned to a red (*n* = 32) or blue (*n* = 32) condition. Participants entered a soundproof laboratory with comfortable light and were told that the purpose of the experiment was to test their maximum handgrip force. The experimental task included two handgrip force tests. Following instructions provided by the research assistant, participants performed a 5-min arm warm-up. After the warm-up, they were presented with a grip dynamometer, shown how to operate it, and were allowed to get acquainted with it.

The research assistant recorded participant performance on the first handgrip task as a baseline measure. Next, participants were given a piece of red or blue paper, on which they completed a brief demographics questionnaire and two items relating to anxiety and self-efficacy (as in Experiment 1). The RGB criteria of red (255, 0, and 0) and blue (0, 0, and 255) were applied to the red and blue paper. The HSL criteria were as follows: red (0, 240, and 120) and blue (160, 240, and 120). Finally, participants completed the second handgrip force test.

#### Statistical Analysis

Firstly, all data were tested for normal distribution to examine whether they were conformed to normal distribution. Secondly, to compare participant handgrip force test performance between the red condition and blue condition, we performed ANCOVA to control for baseline performance. Thirdly, independent *t*-tests were used to examine anxiety and self-efficacy in the two conditions.

### Results and Discussion

A sample K-S analysis proved that the data was a normal distribution (*Z* = 0.112, *P* = 0.058). ANCOVA revealed a significant effect of color condition, *F*(1, 2) = 5.44, *p* = 0.023, η^2^ = 0.10. Participant performance in the red condition (*M* = 52.83, *SD* = 0.57) was higher than in the blue condition (*M* = 50.94, *SD* = 0.57). This result was consistent with the hypothesis that red rather than blue would enhance gross motor skill (handgrip) performance. This suggests that the motor skill improvement induced by red has a significant effect on strength (gross motor skill) in contrast to Experiment 1 where a positive effect on performance was induced by blue (fine motor skill).

However, independent *t*-tests showed that there were no significant differences in anxiety (*t* = 0.58, *p* = 0.57) or self-efficacy (*t* = 1.41, *p* = 0.16) between the two color conditions. Although this was inconsistent with our hypothesis, it does not mean that viewing red enhanced grip strength independently of arousal. The possible reason is that anxiety and self-efficacy are not strong contributors to performance on this task. Nonetheless, these results revealed that the red condition enhanced participants’ gross motor performance compared to the blue condition.

## General Discussion

The results of Experiment 1 showed that in the fine motor skill condition, dart-throwing performance in the blue context was better than in the red context. Participant anxiety and self-efficacy scores provide an explanation for the relationship between color and dart-throwing performance. Compared with blue, red caused higher anxiety and lower self-efficacy. Thus, the results of Experiment 1 were consistent with our predictions. According to the inverted-U hypothesis, higher arousal is detrimental to fine motor skill performance. In Experiment 1, participants’ anxiety scores were higher and dart-throwing performance was lower in the red condition, consistent with the assertion that red is detrimental to fine motor skill. The results are also consistent with previous research on cognitive tasks demonstrating that high arousal is disadvantageous when completing difficult tasks (Xia et al., 2016). In addition to the relationship between arousal and motor performance, one possible reason that red reduces fine motor skill may also be related to the red-threat link (Elliot and Aarts, 2011). As a threat signal, red could increase participant anxiety and task distraction, meaning that participants need to mobilize mental resources to inhibit distraction and maintain their attention on the task. We suggest that viewing red depletes individuals’ resources for self-control, and this impairs performance on skill-based motor tasks (e.g., dart-throwing). The results of Experiment 1 showed that participants’ anxiety scores were higher in the red condition than in the blue condition. An alternative to the idea that red depletes individuals’ resources for self-control is that the red color increased anxiety, which in turn interfered with coordination and control of dart throwing by increasing muscular tension.

The results of Experiment 2 showed that participants had stronger grip strength in the red context compared to blue. These results are in line with our hypothesis, and also in agreement with the findings of Elliot and Aarts (2011). It is suggested that red could improve gross motor skill performance. As the results of Experiment 2 are consistent with Elliot and Aarts (2011), the red-threat link could explain why red enhanced grip strength. More specifically, the red-threat link activated participants’ avoidance/defense systems, which enabled them to mobilize resources for defense. There were no significant differences in participant anxiety or self-efficacy scores between the red and blue conditions. These null findings for anxiety and self-efficacy do not mean that viewing red enhances grip strength independently of arousal. There are two possible reasons for these null findings. First, in contrast to Experiment 1, the red/blue stimulus (which was the questionnaire) was presented before the grip task in Experiment 2, and the duration of viewing red/blue in Experiment 2 may have been too short. Most participants completed the questionnaire in around 30 s, which means participants were exposed to the red/blue stimulus only for this time. Payen et al. (2011) found no effect of color on general arousal and mood when the duration of red stimulus in their study was about 2 s. Although the duration of viewing red in Experiment 2 is longer than Payen et al. (2011), it may still be difficult to improve participant anxiety levels so quickly. In addition, in contrast to the two grip strength tests, anxiety and self-efficacy were assessed only once. The lack of pretest measurements for anxiety and self-efficacy scores may also lead to the lack of difference between the two groups.

The study by Hill and Barton (2005) showed that combat athletes wearing red uniforms won significantly more often than athletes wearing blue uniforms. Namely, athletes in red had advantages over those in blue. However, this study failed to clarify whether the red uniform reduced the performance of the opponents or improved the performance of the wearers. Later studies confirmed the association between red and aggressiveness (Geng et al., 2021), and found that the red-aggressiveness link affects observers’ subjective judgments (e.g., that of referees) (Hagemann et al., 2008; Krenn, 2014, 2015). Based on the red-aggressiveness link, it seem to suggest that red affect observers’ (referees) judgment of competitive performance, but it not answer the question of how red affect opponents’ motor behavior.

From an applied perspective, the results of Experiments1 could explain the advantage of red uniforms as found by Hill and Barton (2005). There are many movements requiring target hitting in combat sport. The athlete’s aim is to hit opponents accurately (e.g., boxing, tae kwon do). This is similar to dart-throwing, which emphasizes the precision of movement. Red might be distractive when completing fine motor skills because it is associated with threat and high arousal. Thus, red may be detrimental to fine motor skills requiring precision over an extended period of time, as it depletes individuals’ cognitive resources.

However, the results of Experiment 2 show that viewing red before a motor task enhances grip strength, suggesting that red may be beneficial for gross motor skills requiring short bursts of brute force. From this point of view, combat athletes wearing red uniforms would serve to enhance the strength of their opponents (Falcó et al., 2016). It should be noted that, like Elliot and Aarts (2011), the duration of viewing red in Experiment 2 was relatively short. Elliot and Maier (2014) argued that prolonged exposure to a certain color may cause participants to adapt to that color and thus weaken the psychological effect of it. It is unclear whether a longer duration of viewing red still leads to improved gross motor skill performance.

The present study explored the effects of red and blue on different types of motor performance. In addition to comparing the effects of red and blue on cognition and behavior (including motor skills), red and green are often compared (O’Connell et al., 1985; Zhang and Han, 2014; Briki et al., 2015; Krenn, 2015; Hong et al., 2020). Similar to blue, green is also a naturally color with shorter wavelength (Goldstein, 1942). An early study showed that long-wavelength colors may induce feelings of high arousal while short-wavelength colors may induce feelings of low arousal (Walters et al., 1982). Future research should compare the effects of red and green on motor skills, and test whether green has the same effects as blue.

## Limitations

Based on the results of the present study, it can be concluded that performance of fine motor skill (dart-throwing) in the blue condition was better than in the red condition, and performance of gross motor skill (handgrip) in the red context was better than in the blue context. However, the present study has limitations that need to be taken into consideration. Firstly, although the results of the present study are consistent with the inverted U hypothesis, the study only measured anxiety rather than arousal. Arousal is a general physiological and psychological activation of the organism that varies on a continuum from deep sleep to intense excitement (Robert, 2011). In terms of the definition of arousal, arousal includes physiological arousal and psychological arousal. Physiological arousal is a solid foundation for psychological arousal. Psychological arousal is a kind of subjective experience and cognitive evaluation based on physiological arousal. Physiological arousal is typically measured using heart rate, skin conductance, or EEG. Psychological arousal is typically measured by asking participants about their level of excitement, energy, or tension. In addition, psychological arousal can be negative (e.g., anxiety) or positive (e.g., excitement). Anxiety is a negative emotional state in which feelings of nervousness, worry, and apprehension are associated with activation or arousal of the body (Robert, 2011). Therefore, anxiety may be a negative psychological arousal, which is an emotional response that can be triggered by arousal and can also increase arousal. Obviously, anxiety cannot be equated with arousal, and it is a part of arousal. As the study lacked a direct measure of arousal, this may be the reason why the study did not find anxiety as a modulating factor that affects grip strength performance. Secondly, all participants were males, there is evidence about differences between males and females regarding color effect (Falcó et al., 2016). Therefore, further research should include more subjects, such as females. Finally, the methodology conducted in two experiments was different. In Experiment 1, a within-subjects design was used to expose participants to the different colored walls during dart-throwing. However, in Experiment 2, the participants were exposed to the two different colors in a between-subjects design. Moreover, the timing and duration of exposure as well as the size of the colored stimulus was different in the two experiments. Consequently, it may be inappropriate to make comparisons across the two experiments. Indeed, the measures of anxiety and self-efficacy were quite crude in present study. Future research should adopt more rigorous methods to explore this issue. In order to increase ecological validity, future research should also conduct a more ecologically valid approach.

## Conclusion

Our experiments identified differential influences of red and blue on two types of motor skill. More specifically, red was associated with poorer performance than blue on dart throwing whereas red was associated with better performance than blue on a hand grip task.

## Data Availability Statement

The raw data supporting the conclusions of this article will be made available by the authors, without undue reservation.

## Ethics Statement

The studies involving human participants were reviewed and approved by the WSU Medical Ethics Committee. The patients/participants provided their written informed consent to participate in this study.

## Author Contributions

YG was responsible for the design of the study. XH and AX were responsible for data collection and for writing the article. YS and LG analyzed data and revised the article. RZ assisted in the experiment. All authors have approved the manuscript and given consent for its submission and subsequent publication.

## Conflict of Interest

The authors declare that the research was conducted in the absence of any commercial or financial relationships that could be construed as a potential conflict of interest.

## Publisher’s Note

All claims expressed in this article are solely those of the authors and do not necessarily represent those of their affiliated organizations, or those of the publisher, the editors and the reviewers. Any product that may be evaluated in this article, or claim that may be made by its manufacturer, is not guaranteed or endorsed by the publisher.

## References

[B1] Attrill M. J. Gresty K. A. Hill R. A. Barton R. A. (2008). Red shirt colour is associated with long-term team success in English football. *Int. J. Sports Sci.* 26 577–582. 10.1080/02640410701736244 18344128

[B2] Briki W. Rinaldi K. Riera F. Trong T. T. Hue O. (2015). Perceiving red decreases motor performance over time: a pilot study. *Eur. Rev. Appl. Psychol*. 65 301–305. 10.1016/j.erap.2015.09.001

[B3] Bruno N. Martani M. Corsini C. Oleari C. (2013). The effect of the color red on consuming food does not depend on achromatic (Michelson) contrast and extends to rubbing cream on the skin. *Appetite* 71 307–313. 10.1016/j.appet.2013.08.012 23999521

[B4] Cohen J. (ed.) (1988). *Statistical Power Analysis for the Behavioral Sciences*, 2th Edn. Hillsdale: Lawrence Erlbaum.

[B5] Crane D. K. Hensarling R. W. Jung A. P. Sands C. D. Petrella J. K. (2008). The effect of light color on muscular strength and power. *Percept. Mot. Skills* 106 958–962. 10.2466/pms.106.3.958-962 18712217

[B6] Curby D. G. (2016). Effect of uniform color on outcome of match at Senior World Wrestling Championships 2015. *Int. J. Wrestl. Sci.* 6 62–64. 10.1080/21615667.2016.1210266

[B7] Dreiskaemper D. Strauss B. Hagemann N. Büsch D. (2013). Influence of red jersey color on physical parameters in combat sports. *J. Sport Exerc. Psychol.* 35 44–49. 10.1080/00140139.2013.774833 23404878

[B8] Elliot A. J. Aarts H. (2011). Perception of the color red enhances the force and velocity of motor output. *Emotion* 11 445–449. 10.1037/a0022599 21500913

[B9] Elliot A. J. Maier M. A. (2014). Color psychology: effects of perceiving color on psychological functioning in humans. *Annu. Rev. Psychol.* 65 95–120. 10.1146/annurev-psych-010213-115035 23808916

[B10] Elliot A. J. Niesta D. (2008). Romantic red: red enhances men’s attraction to women. *J. Pers. Soc. Psychol.* 95 1150–1164. 10.1037/0022-3514.95.5.1150 18954199

[B11] Falcó C. Conchado A. Estevan I. (2016). Theeffect of color on the use of electronic body protectors in taekwondo matches. *Percept. Mot. Skills* 122 812–824. 10.1177/0031512516649958 27287051

[B12] Geng L. Hong X. Zhou Y. (2021). Exploring the implicit link between red and aggressiveness as well as blue and agreeableness. *Front. Psychol.* 11:570534. 10.3389/fpsyg.2020.570534 33519586 PMC7844062

[B13] Goldstein K. (1942). Some experimental observations concerning the influence of colors on the function of the organism. *Am. J. Phys. Med. Rehabil.* 21 147–151. 10.1097/00002060-194206000-00002

[B14] Green W. K. Hasson S. M. Mohammed S. K. Phillips C. L. White A. (1982). Effect of viewing selected colors on the performance of gross and fine motor tasks. *Percept. Mot. Skills* 54:778. 10.2466/pms.1982.54.3.778 7099886

[B15] Guéguen N. Jacob C. Lourel M. Pascual A. (2012). When drivers see red: car color frustrators and drivers’ aggressiveness. *Aggress. Behav.* 38 166–169. 10.1002/ab.21416 25363640

[B16] Hagemann N. Strauss B. Leissing J. (2008). When the referee sees red. *Psychol. Sci.* 19 769–771. 10.1111/j.1467-9280.2008.02155.x 18816283

[B17] Hill R. A. Barton R. A. (2005). Red enhances human performance in contests. *Nature* 435:293. 10.1038/435293a 15902246

[B18] Hong X. He Z. Zou R. (2020). Individual differences in implicit associations between red and green colors and failure and success among Chinese adults. *Soc. Behav. Pers.* 48:e9228. 10.2224/sbp.9228

[B19] Ingram F. Lieberman L. R. (1985). Effects of expectations on the performance of hand grip after viewing selected hues. *Percept. Mot. Skills* 61:370. 10.2466/pms.1985.61.2.370 4069902

[B20] Jacobs K. W. Suess J. F. (1975). Effects of four psychological primary colors on anxiety state. *Percept. Mot. Skills* 41 207–210. 10.2466/pms.1975.41.1.207 1178407

[B21] Jiang F. Lu S. Yao X. Yue X. Au W. (2014). Up or down? How culture and color affect judgments. *J. Behav. Decis.Mak.* 27 226–234. 10.1002/bdm.1800

[B22] Krenn B. (2014). The impact of uniform color on judging tackles in association football. *Psychol. Sport Exerc.* 15 222–225. 10.1016/j.psychsport.2013.11.007

[B23] Krenn B. (2015). The effect of uniform color on judging athletes’ aggressiveness, fairness and chances of winning. *J. Sport Exerc. Psychol.* 37 207–212. 10.1123/jsep.2014-0274 25996111

[B24] Mehta R. Zhu R. (2009). Blue or red? Exploring the effect of color on cognitive task performances. *Science* 323 1226–1229. 10.1126/science.1169144 19197022

[B25] O’Connell B. J. Harper R. S. McAndrew F. T. (1985). Grip strength as a function of exposure to red or green visual stimulation. *Percept. Mot. Skills* 61 1157–1158. 10.2466/pms.1985.61.3f.1157 4094856

[B26] Oxendine J. B. (1970). Emotional arousal and motor performance. *Quest Monogr.* 13 23–32. 10.1080/00336297.1970.10519673

[B27] Payen V. Elliot A. J. Coombes S. A. Chalabaev A. Cury F. (2011). Viewing red prior to a strength test inhibits motor output. *Neurosci. Lett.* 495 44–48. 10.1016/j.neulet.2011.03.032 21406218

[B28] Piatti M. Savage D. A. Torgler B. (2012). The red mist? Red shirts, success and team sports. *Sport Soc.* 15 1209–1227. 10.1080/17430437.2012.690400

[B29] Profusek P. J. Rainey D. W. (1987). Effects of baker-miller pink and red on state anxiety, grip strength, and motor precision. *Percept. Mot. Skills* 65 941–942. 10.2466/pms.1987.65.3.941 3438141

[B30] Robert S. W. (ed.) (2011). *Foundation of Sport and Exercise Psychology*, 5th Edn. Champaign: Human Kinetics.

[B31] Sorokowski P. Szmajke A. (2011). The influence of the “red win” effect in sports: a hypothesis of erroneous perception of opponents dressed in red-preliminary test. *Hum. Mov.* 12 367–373. 10.2478/v10038-011-0043-5

[B32] Stone N. J. (2003). Environmental view and color for a simulated telemarketing task. *J. Environ. Psychol.* 23 63–78. 10.1016/S0272-4944(02)00107-X

[B33] Walters J. Apter M. J. Svebak S. (1982). Color preference, arousal, and the theory of psychological reversals. *Motiv. Emot.* 6 193–215. 10.1007/BF00992245

[B34] Wexner L. B. (1954). The degree to which colors (hues) are associated with mood-tones. *J. Appl. Psychol.* 38 432–435. 10.1037/h0062181

[B35] Williams J. M. (1994). *Applied Sport Psychology: Personal Growth to Peak Performance*, 3rd Edn. California: Mayfield Publishing Company Press.

[B36] Wright B. Rainwater L. (1962). The meaning of color. *J. Gen. Psychol.* 67 89–99. 10.1080/00221309.1962.9711531 14008415

[B37] Xia T. Song L. Wang T. T. Tan L. Mo L. (2016). Exploring the effect of red and blue on cognitive task performances. *Front. Psychol.* 7:784. 10.3389/fpsyg.2016.00784 27303343 PMC4880552

[B38] Yerkes R. M. Dodson J. D. (1908). The relation of strength of stimulus to rapidity of habit formation. *J. Comp. Neurol. Psychol*. 18 459–482. 10.1002/cne.920180503

[B39] Zhang T. Han B. (2014). Experience Reverses the Red Effect among Chinese Stockbrokers. *PLoS One* 9:e89193. 10.1371/journal.pone.0089193 24586587 PMC3933460

